# Infection with HIV and HCV enhances the release of fatty acid synthase into circulation: evidence for a novel indicator of viral infection

**DOI:** 10.1186/1471-230X-10-92

**Published:** 2010-08-13

**Authors:** Gerard Aragonès, Carlos Alonso-Villaverde, Cristina Oliveras-Ferraros, Raúl Beltrán-Debón, Anna Rull, Fernando Rodríguez-Sanabria, Jordi Camps, Alejandro Vázquez Martín, Javier A Menéndez, Jorge Joven

**Affiliations:** 1 Centre de Recerca Biomèdica, Hospital Universitari de Sant Joan, Institut d'Investigació Sanitària Pere Virgili (IISPV), Universitat Rovira i Virgili, Reus, Spain; 2 Servei de Medicina Interna, Hospital Son Llàtzer, Palma, Illes Balears, Spain; 3 Catalan Institute of Oncology (ICO), Dr. Josep Trueta. University Hospital, Girona, Spain; 4 Biomedical Research Institute (IdIBGi), Dr. Josep Trueta. University Hospital, Girona, Spain

## Abstract

**Background:**

Fatty acid synthase (FASN) is an enzyme synthesized by the liver and plays an important role in lipogenesis. The present study aimed to investigate whether serum FASN concentration may provide a direct link between HIV and/or HCV viral infections and lipid metabolic disorders commonly observed in HIV/HCV-infected patients.

**Methods:**

We evaluated serum FASN concentration in 191 consecutive HIV-infected patients in the absence or presence of HCV co-infection. For comparison, 102 uninfected controls were included. Metabolic and inflammatory phenotype was also compared with respect to the presence of HCV co-infection.

**Results:**

Serum FASN concentration was significantly higher in HIV-infected patients than in healthy participants and HCV co-infected patients showed higher levels than those without co-infection. Levels were also affected by treatment regimen, but marginally influenced by virological variables. Insulin concentration was the sole variable among metabolic parameters that demonstrated a significant correlation with serum FASN concentrations. Serum alanine aminotransferase (ALT) values correlated significantly with serum FASN concentration and provided the best discrimination with respect to the presence or absence of HCV co-infection. In multivariate analysis, only ALT, monocyte chemoattractant protein-1 (MCP-1) and the presence of antiretroviral treatment regimen significantly contributed to explain serum FASN concentration in HIV/HCV co-infected patients.

**Conclusion:**

Serum FASN concentration is significantly increased in HIV-infected individuals. The release of FASN into the circulation is further enhanced in patients who are co-infected with HCV. Subsequent studies should explore the usefulness of this indicator to monitor the effect of viral infections on disease progression and survival.

## Background

The life expectancy of HIV-infected patients has dramatically improved with the use of antiretroviral therapies. Unfortunately, the spectrum of metabolic alterations and concurrent diseases, especially cardiovascular complications, is being expanded gradually and more than 10% of HIV-infected patients experience life-threatening clinical manifestations that could be treated and/or prevented [1]. Although HIV-related metabolic alterations have been attributed, at least in part, to the use of antiretroviral treatments, both dyslipidemia and insulin resistance can also be observed in treatment-naïve HIV-infected patients suggesting a metabolically deleterious role for the HIV infection itself [2,3].

The mechanism by which HIV alters lipid metabolism in an infected cell is an issue largely unresolved; however, it was recently reported that HIV replication can significantly alter the expression of key cellular proteins closely involved in the maintenance of normal metabolic pathways [4,5]. Proteomic studies by Chan *et al *[4] initially reported upregulation of Fatty Acid Synthase (FASN) after HIV infection of cultured cells *in vitro*. Their findings causally associated this lipogenic enzyme - which remained undetectable in uninfected control cells - to the deregulation of the cell cycle after the HIV infection. Rasheed *et al *[5] further confirmed and expanded the notion that HIV replication alone (*i.e. *without any influence of antiviral drugs, or other human genetic factors) notably and specifically enhances production of FASN and other important proteins that mostly participate in lipid metabolism. FASN is also significantly up-regulated during infection with HCV [6,7], a frequent co-infection in HIV-patients [8]. We have therefore hypothesized that measurement of circulating FASN may provide a direct link between HIV and/or HCV viral infections and lipid metabolic disorders commonly observed in HIV/HCV-infected patients.

FASN, a 250-kDa cytosolic multi-enzyme that is the major responsible for the *de novo *lipogenesis, functions normally in the liver whereas it is minimally expressed in most other normal human tissues [9]. FASN overexpression and the subsequent change in cell lipogenic phenotype may therefore be crucial in determining the fate of such cell in response to different stimuli. This notion has been already documented in human cancer pathogenesis. FASN is highly expressed in most human cancers and its inhibition leads to selective apoptotic death of cancer cells both *in vitro *and *in vivo *[10]. Cultured cancer cells can excrete immunoreactive FASN into the extracellular space and the detection of significant amounts of FASN in the blood of patients with breast, prostate, and ovarian cancer may also represent a potential biomarker for human cancer because serum FASN concentration is further increased in the late (metastatic) stages of human malignancies [11-14]. Although the ultimate mechanism regulating the extracellular release of FASN remains largely undefined [14,15], we might envisage that serum FASN concentrations would become significantly altered in HIV-infected patients with respect to healthy controls and this could be related to the presence of HCV co-infection.

## Methods

### Participants

Patients included in this study attended the Hospital Universitari de Sant Joan in Reus, Spain. We performed a cross-sectional study in 191 consecutive HIV-infected patients who accepted the invitation to participate in the study and provided fully informed consent. Criteria for inclusion consisted of age greater than 18 years, no pre-existent AIDS-related opportunistic diseases and the absence of acute disease or clinical signs of inflammation. Patients with clinical or analytical evidence of renal insufficiency, cancer, neurological disorders or major liver damage were excluded. To avoid any possible bias, inclusion in the study was evaluated by personnel not directly responsible for these patients. The study was approved by the local Ethics Committee in accordance with the Declaration of Helsinki. For comparisons of selected variables we included a general population-based control group of unrelated subjects (n = 102), free of HIV and HCV infection or any other inflammatory or metabolic alteration. Further details of the control group have been described elsewhere [16].

### Clinical and laboratory studies

Detailed clinical characteristics of each subject were recorded and a thorough physical examination was performed during the interview. We recorded data regarding HIV/HCV infections including opportunistic infections, mode of HIV transmission, CD4 cell count and HIV/HCV viral loads, HCV genotypes, and details of the current and past antiretroviral therapy. Lipodystrophy was defined as the presence of body-fat changes that included subcutaneous lipoatrophy (hollow cheeks, prominent superficial veins in the limbs or flattening of the buttocks) and/or lipohypertrophy (central obesity, breast enlargement or dorsocervical fat pad). Data concerning classical cardiovascular risk factors and laboratory variables were also included. Blood pressure was determined according to standardized methods. Body-mass index was defined as weight (kg)/height (m^2^). Fasting serum glucose, insulin, C-reactive protein, apolipoprotein A I, cholesterol, non-esterified fatty acids (NEFAs) and triglycerides were measured with the automatic analyzer LXi 725-Synchron (Beckman Coulter, Fullerton, California, USA) using enzymatic assays or chemiluminescent immunoassays. HDL-cholesterol and LDL-cholesterol levels were measured as described [17,18]. CD4+ and CD8+ T cells were counted using FACscan flow cytometry (Becton Dickinson, Madrid, Spain). Interleukin-6 (IL-6), interleukin-8 (IL-8) and monocyte chemoattractant protein-1 (MCP-1) were measured by a multiplex cytometric bead-based assay (FlowCytomix Multiplex; BenderMedsystems, Vienna, Austria) and were analyzed in an EPICS-XL-MCL flow cytometer (Beckman-Coulter, Fullerton, CA) following the manufacturer's protocol. Circulating levels of the extracellular form of FASN were measured by ELISA (FASgen Inc., Baltimore, MA).

### Statistical analysis

Results are expressed as the mean (S.E.M) unless otherwise indicated. The Kolmogorov-Smirnov test was applied to check the normality of the variables. ANOVA was used to test the differences in continuous variables and the chi-square test was used for categorical variables. Non-parametric tests were used when appropriate. We performed multiple linear regression of FASN as a dependent variable; included in the model were classical variables and those variables for which the univariable analysis showed a *p*-value of at least 0.1 and collineality was assessed. All reported *p*-values are two-tailed and values greater than 0.05 denote statistical significance. The SPSS 17.0 package was used to perform the statistical analysis.

## Results

Most HIV-infected patients in this study were smokers (80.6%), 127 (66.5%) were male, and their ages ranged from 29 years to 64 years. 114 (59.7%) had undetectable HIV-1 viral load. The predominant cause of infection was intravenous drug abuse (59.2%) and in the remaining patients, sexual factors were positively identified. Mean time since first serologic evidence of HIV infection was 4.29 (0.3) years. Obesity was not found in these patients but 47 (24.1%) showed severe lipodystrophy. 115 (60.2) patients were co-infected with HCV. 64 patients were treated with protease inhibitor (PI)-containing regimen, 76 with non-nucleoside analogues reverse transcriptase inhibitors (NNRTI)-based therapy, and 51 currently not receiving any type of antiretroviral therapy for at least 6 months (Table 1).

**Table 1:** Demographic and clinical characteristics of HIV-infected patients (*n *= 191)

Characteristics	Values
**Age, years**	38.8 (0.51)
**Gender, male (%)**	127 (66.5)
**BMI, kg/m**^**2**^	22.93 (0.26)
**Current smokers, n (%)**	154 (80.6)
**Lipodystrophy, n (%)**	47 (24.1)
**Time of sero-prevalence, years**	4.29 (0.3)
**Risk factors for HIV, n (%)**	
Intravenous drug users	113 (59.2)
Heterosexual contact	54 (28.3)
Male homosexual contact	24 (12.5)
**Treatment scheme, n (%)**	
Untreated	51 (26.7)
Non-nucleoside analogues	76 (39.8)
Protease inhibitors	64 (33.5)
**Undetectable HIV-1 viral load**^**a**^**, n (%)**	114 (59.7)
**CD4+ T lymphocyte count, cells/mL**	459 (22.8)
**HCV co-infection, n (%)**	115 (60.2)
**HCV genotypes, %**	
I	78.3
II	3.8
III	14.9
IV	3.0
**Log-HCV viral load, copies/mL**	5.89 (1.14)

Values are the mean and the standard errors of the mean (S.E.M.), unless otherwise indicated.

^a ^Limit of detection: 40 copies/mL.

**Figure 1:**
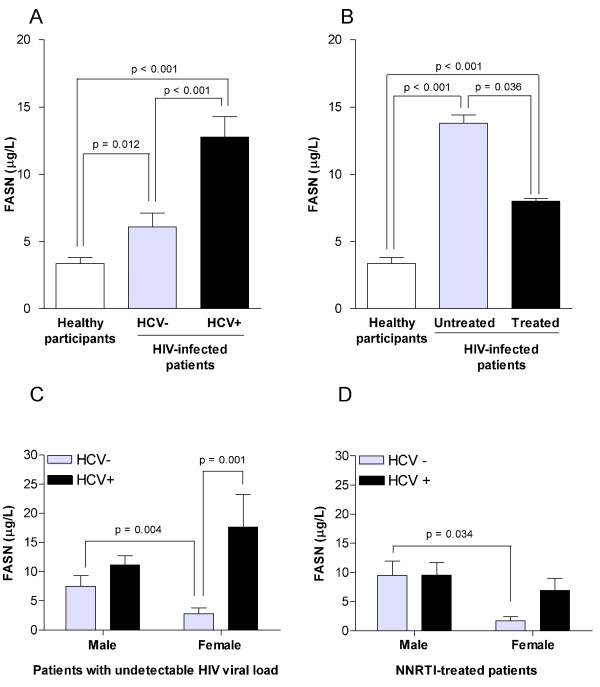
**Circulating levels of the extracellular form of FASN**. Distribution of serum FASN concentrations according to the presence of antiretroviral treatment (A) or to the presence of HCV co-infection (B) in HIV-infected patients and in healthy participants; gender differences were only appreciable in patients with undetectable HIV-1 viral load (C) or in those undergoing the NNRTI-based antiretroviral treatment regimen (D).

Circulating levels of the extracellular form of FASN were systematically and significantly higher in HIV-infected patients than in healthy participants. Of note, co-infection with HCV further significantly increased serum FASN concentration (Figure 1A). Moreover, circulating FASN levels were significantly affected by the presence of antiretroviral treatment. As shown in Figure 1B, untreated HIV-infected patients had higher levels of circulating FASN than those patients under antiretroviral therapy. However, serum FASN levels were marginally affected by the type of antiretroviral treatment. Both in the healthy control group and in the HIV-infected group we failed to observe significant associations between age or gender and serum FASN concentrations. However, a significant association was found in women with undetectable HIV viral load when HIV-infected patients were segregated according to the presence of HCV co-infection (Figure 1B). Gender-based differences in serum FASN concentration were also observed in patients undergoing an NNRTI-based antiretroviral treatment regimen (Figure 1C).

We then examined the metabolic phenotype in participants according to immunological and virologic variables such as HIV/HCV viral loads as well as HCV genotype (Tables 2 and 3). Serum FASN concentration was not influenced by any virologic variable considered here but there was a significant (p = 0.02) correlation (ρ = 0.28) between serum FASN concentration and lymphocyte T CD4+ count but only in those patients with detectable HIV viral load, suggesting that enhanced levels of extracellular FASN might come, at least in part, from lymphocyte T CD4+ T cells with active viral replication. Patients with lipodystrophy, in which distribution of HCV co-infected patients was identical to that of the whole group, also showed similar serum FASN concentrations [9.7 (3.2) μg/L] with respect to patients without such condition [10.4 (4.1) μg/L]. There was also a trend towards higher HOMA-IR values in HCV co-infected patients which was significantly altered by NNRTI-regimen treatment and that positively correlated with serum FASN concentrations. HIV infection caused a significant and deleterious effect on HDL-cholesterol, apolipoprotein AI and triglycerides concentrations irrespective of HCV co-infection and treatment regimen in comparison with the healthy group. However, we failed to identify statistically significant changes in serum NEFAs concentrations among patients with HCV co-infection or among patients without co-infection.

**Table 2:** Metabolic profile of healthy participants and HIV-infected patients segregated according to HCV co-infection and HIV-1 viral load

		HIV-infected patients			
		
	Control group	Non-HCV co-infected group		HCV co-infected group	
			
	(*n *= 102)	VL < 40 copies/mL	VL > 40 copies/mL	VL < 40 copies/mL	VL > 40 copies/mL
		(n = 47)	(n = 29)	(n = 67)	(n = 48)
**FASN, μg/L**	3.36 (0.92)	5.83 (1.34) ^a^	5.08 (1.74) *	12.69 (2.01) ^c^	14.41 (2.91) ^c^
**NEFA, mmol/L**	0.65 (0.04)	0.62 (0.06)	0.64 (0.06)	0.63 (0.04)	0.61 (0.05)
**Glucose, mmol/L**	4.96 (0.16)	5.61 (0.20) ^c^	5.2 (0.14) ^b^	5.42 (0.12) ^c^	5.16 (0.10) ^b^
**Insulin, pmol/L**	64.6 (6.59)	69.22 (9.68)	47.95 (6.11) ^a,^*	84.40 (10.79) ^a^	76.31 (11.82)
**HOMA-IR**	1.15 (0.20)	1.33 (0.18)	0.92 (0.14) ^a,^*	1.64 (0.22) ^a^	1.48 (0.25)
**Cholesterol, mmol/L**	5.27 (0.12)	5.52 (0.19)	5.14 (0.23)	4.74 (0.15) ^c^	4.41 (0.16) ^c^
**HDL-cholesterol, mmol/L**	1.36 (0.03)	1.24 (0.09) ^a^	1.15 (0.08) ^b^	1.21 (0.05) ^b^	1.07 (0.05) ^c, ^*
**LDL-cholesterol, mmol/L**	3.19 (0.79)	3.28 (0.17)	2.96 (0.17)	2.71 (0.13) ^c^	2.38 (0.13) ^c^
**Triglyceride, mmol/L**	1.27 (0.9)	3.00 (0.47) ^c^	3.11 (1.01)	2.04 (0.18) ^c^	3.24 (1.09) ^c^
**Apolipoprotein AI, g/L**	1.69 (0.05)	1.44 (0.05) ^c^	1.26 (0.04) ^c, ^**	1.40 (0.03) ^c^	1.33 (0.04) ^c^
**Alanine aminotransferase, μKat/L**	0.46 (0.02)	0.45 (0.03)	0.51 (0.04)	0.98 (0.11) ^c^	0.97 (0.11) ^c^
**Aspartate aminotransferase, μKat/L**	0.41 (0.02)	0.41 (0.03)	0.47 (0.08)	0.88 (0.09) ^c^	0.88 (0.99) ^c^

Values are the mean and the standard error of the mean (S.E.M.).

VL indicates HIV-1 viral load; HOMA-IR is homeostasis model assessment-estimated insulin resistance; NEFA is non-esterificated fatty acids.

^a ^P < 0.05, ^b ^P < 0.01 and ^c ^P < 0.001 with respect to control group; * P < 0.05 and ** P = 0.006 with respect to patients with VL < 40 copies/mL in the corresponding group

**Table 3:** Metabolic profile of HIV-infected patients segregated according to HCV co-infection and antiretroviral therapy

	Non-HCV co-infected group			HCV co-infected group		
		
	Untreated	NNRTI	PI	Untreated	NNRTI	PI
	(*n *= 19)	(*n *= 32)	(*n *= 25)	(*n *= 32)	(*n *= 44)	(*n *= 39)
**FASN, μg/L**	5.14 (2.10)	7.40 (1.81)	4.15 (1.00)	20.01 (4.0)	8.64 (1.51) ^b^	11.75 (2.41) ^a^
**NEFA, mmol/L**	0.53 (0.07)	0.63 (0.07)	0.77 (0.1)	0.65 (0.06)	0.67 (0.05) *	0.52 (0.04)
**Glucose, mmol/L**	5.21 (0.20)	5.77 (0.29) *	5.09 (0.09)	5.13 (0.13)	5.62 (0.19) ^a^	5.21 (011)
**Insulin, pmol/L**	41.91 (7.1)	70.74 (13.4) ^a^	62.23 (8.6) ^a^	71.35 (11.2)	103.2 (19.8)	72.24 (10.7)
**HOMA-IR**	0.80 (0.16)	1.38 (0.27) ^a^	1.14 (0.16)	1.41 (0.25)	2.06 (0.42)	1.36 (0.21)
**Cholesterol, mmol/L**	4.59 (0.24)	5.34 (0.23) ^a,^*	6.10 (0.28) ^c^	4.41 (0.19)	4.67 (0.23)	4.7 (0.16)
**HDL-cholesterol, mmol/L**	1.18 (0.09)	1.30 (0.12)	1.18 (0.10)	1.17 (0.07)	1.20 (0.05) *	1.09 (0.6)
**LDL-cholesterol, mmol/L**	2.69 (0.22)	3.10 (0.20) *	3.59 (0.23) ^a^	2.57 (0.15)	2.54 (0.18)	2.70 (1.15)
**Triglyceride, mmol/L**	1.57 (0.29)	2.60 (0.44) ^a^	4.87 (1.50) ^b^	2.99 (1.71)	2.35 (0.36) ^a^	2.17 (0.22) ^b^
**Apolipoprotein AI, g/L**	1.39 (0.06)	1.43 (0.07)	1.34 (0.06)	1.33 (0.06)	1.45 (0.04) *	1.32 (0.04)
**Alanine aminotransferase, μKat/L**	0.52 (0.10)	0.57 (0.11)	0.45 (0.06)	1.16 (0.16)	0.91 (0.11)	0.69 (0.07) ^a^
**Aspartate aminotransferase, μKat/L**	0.45 (0.07)	0.45 (0.04)	0.41 (0.08)	1.02 (0.13)	0.94 (0.14)	0.69 (0.07) ^a^

Values are the mean and the standard error of the mean (S.E.M.).

HOMA-IR is homeostasis model assessment-estimated insulin resistance; NEFA indicates non-esterificated fatty acids; NNRTI: non-nucleoside analogues reverse transcriptase inhibitors; PI: protease inhibitors;

^a ^P < 0.05, ^b ^P < 0.01 and ^c ^P < 0.001 vs untreated patients of the corresponding group; * P < 0.05 with respect to PI-treated patients in the corresponding group

**Figure 2:**
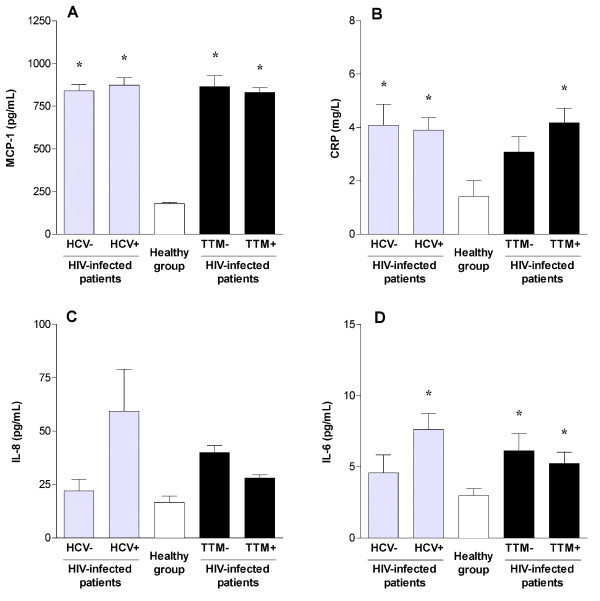
**Circulating levels of pro-inflammatory biomarkers**. Distribution of serum MCP-1 (A), C-reactive protein (B), interleukin-8 (C) and interleukin-6 (D) concentrations in healthy subjects and HIV-infected patients segregated according to the presence of HCV co-infection and antiretroviral therapy. TTM-indicates untreated patients; TTM+ indicates treated patients; * indicates p < 0.05.

**Figure 3:**
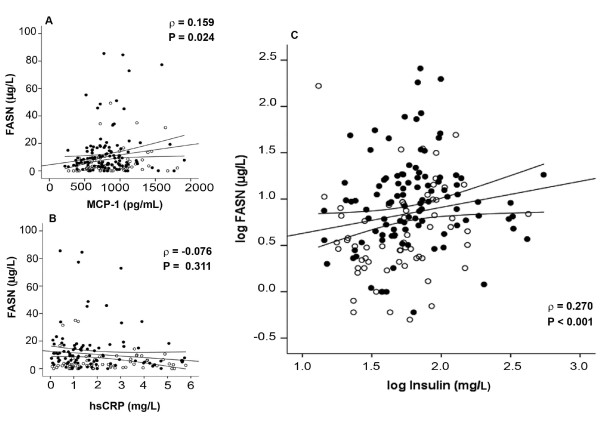
**Relationship between FASN concentration, inflammation and metabolism**. Correlation between serum FASN concentration and MCP-1 (A), C-reactive protein (B) and insulin (C) concentrations in HIV-infected patients. (*Opened circles *indicate non-HCV co-infected patients; and *closed circles *indicate HCV co-infected patients).

Assessed inflammatory biomarkers were consistently elevated in HCV co-infected patients with respect to healthy participants, although the differences were not statistically significant for serum IL-8. Serum MCP-1 and CRP concentrations were significantly increased in HIV-infected patients irrespective to antiretroviral treatment, while co-infection did not result in further differences (Figure 2A and 2B). We could not discriminate serum IL-8 and IL-6 concentrations of the control group from those of non-co-infected patients (Figure 2C and 2D). We also found significant correlations between serum MCP-1 and serum FASN concentrations that were not evident for the serum CRP concentration (Figure 3A and 3B). However, serum MCP-1 concentration was not correlated with any virological parameter above mentioned.

**Figure 4:**
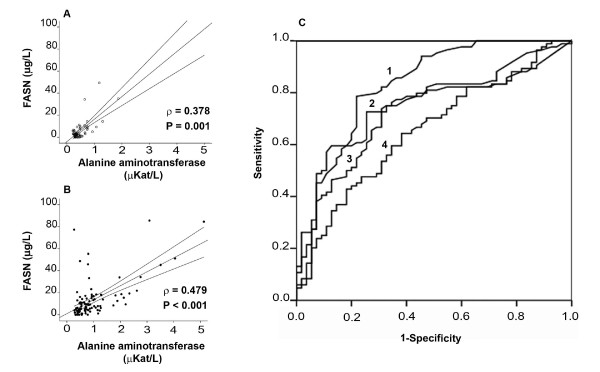
**Relationship between FASN concentration and liver disease**. Correlation between serum FASN concentration and alanine aminotransferase activity both in non-HCV co-infected (A) and HCV co-infected (B) HIV-infected patients. Analyses for the discrimination of HCV co-infected patients using the receiver-operating curves (C) for serum (1) alanine aminotransferase activity, (2) aspartate aminotransferase activity, (3) FASN concentration, and (4) MCP-1 concentrations showed that the relative contributions of FASN and MCP-1 were significant but negligible. The area under the curve values (95% CI), were respectively: 0.834 (0.764-0.904), 0.754 (0673-0.835), 0.713 (0.626-0.801) and 0.652 (0.560-0.744).

Among variables measured to ascertain the metabolic phenotype, only the serum insulin concentration showed a significant correlation with serum FASN, an association that was significant in both co-infected and non-co-infected patients (Figure 3C). As expected, serum aminotransferases were significantly higher in HCV co-infected patients, suggesting possible hepatic cellular leakage or damage (Table 2). Moreover, serum alanine aminotransferase (ALT) values provided the best discrimination with respect to the presence or absence of HCV co-infection and correlated significantly with serum FASN concentration irrespective of the condition of co-infection (Figure 4). In multivariate analysis only ALT, MCP-1 and the presence of treatment regimen significantly contributed to explain serum FASN concentration in HIV-patients co-infected with HCV. This relationship, however, was not maintained in patients not infected by HCV (Table 4).

**Table 4:** Multivariate analyses of the determinants of FASN concentrations in HCV co-infected patients (*n *= 115)

	β Coefficient	95% CI	*P *Values
**Age, years**	0.144	-0.409 to 1.327	0.292
**Gender, male**	0.134	-15.86 to 5.14	0.327
**BMI, kg/m**^**2**^	0.086	-0.75 to 1.677	0.444
**Patients treated with PI or NNRTI, n**	-0.295	-23.92 to 1.56	0.041
**Lipodystrophy, n**	-0.202	-18.147 to 2.19	0.121
**CD4+ T cell count, cells/mm**^**3**^	0.121	-0.005 to 0.19	0.260
**Undetectable HIV-1 viral load**^**a**^**, n**	0.053	-6.559 to 10.55	0.639
**HDL-cholesterol, mmol/L**	0.046	-12.89 to 17.55	0.759
**Triglyceride, mmol/L**	0.050	-2.45 to 3.49	0.726
**Apolipoprotein A-I, g/L**	0.230	-5.063 to 36.14	0.135
**MCP-1, pg/mL**	0.271	2.403 to 0.021	0.021
**Insulin, pmol/L**	-0.009	-0.040 to 0.037	0.937
**IL-8, ng/L**	0.025	-0.012 to 0.016	0.828
**hsCRP, mg/L**	-0.108	-1.00 to 0.328	0.312
**Alanine aminotransferase, μKat/L**	0.599	5.154 to 18.48	0.001
**Aspartate aminotransferase, μKat/L**	-0.073	-9.98 to 6.36	0.668

**Dependent variable**: FASN concentration (μg/L)

BMI: body-mass index; NNRTI: non-nucleoside analogues reverse transcriptase inhibitors; PI: protease inhibitors, hsCRP: high sensitive C-reactive protein.

^a^Limit of detection was 40 copies/mL

## Discussion

It is currently considered that during metastasic progression of human malignancies the intracellular FASN is up-regulated and over-expressed and the excess is finally released from the cytosol to the circulation in a stage-related manner [11-14]. Using a proteomics approach, it has been demonstrated that FASN is significantly up-regulated during both HIV infection and HCV infection [4,5,7]. Our data confirm and extend further these *in vitro *findings in a clinical setting revealing for the first time that HIV-infected patients show significant elevations in circulating serum FASN concentration with respect to healthy uninfected donors. Moreover, the increase in serum FASN concentration is even greater in HIV-patients co-infected with HCV. FASN, an intracellular protein, appears to exit cells during HIV infection and this release into the circulation is exacerbated in the presence of HCV co-infection, a condition that is frequently observed in HIV-infected patients. Based on the gender dependencies that we observed (*i.e. *women with undetectable HIV viral load or in those undergoing NNRTI-based antiretroviral regimen), and considering that women infected with HIV have a higher rate of gonadal dysfunction [19], it could be argued that enhanced levels of serum FASN might arise, at least in part, from stimulatory effects of estrogen on *FASN *gene expression which, in turn, will result in enhanced release of FASN protein [20-22]. Conversely, and perhaps surprisingly, we did not observe any significant relationship between circulating levels of the extracellular form of FASN and HIV-related dyslipidemia and lipodystrophy, two conditions in which enhanced endogenous fatty acid synthesis is closely linked to the accumulation of lipids and disproportionate distribution of tissue-associated fats. Although cytosolic FASN, by the coordinated action of its seven active sites, catalyzes all of the necessary reactions in the synthesis of palmitate [23-25], we failed also to identify statistically significant changes in serum NEFAs concentrations in both groups of HIV-infected patients, with and without HCV co-infection. Therefore, our findings support a model in which, upon infection with HIV and/or HCV, changes in circulating levels of extracellular FASN take place through molecular mechanisms likely unrelated to established pathways that regulate the intracellular FASN expression [9,10,23-25]. Notably, integration of HIV into the host cell chromosome occurs preferentially within genes. Particularly, insulin receptor (IRS), 19p13.3-13.2, is one of the identified integration sites [26] and the knockdown of this gene results in a significant up-regulation of FASN [27]. It is plausible, therefore, that increased levels of serum FASN are not a mere epiphenomenon related to cell destruction and leakage but rather to a pathophysiologic response occurring during the viral infection.

In multivariate analysis, serum MCP-1, but not other inflammatory biomarkers, significantly contributed to explain serum FASN concentration in HCV co-infected patients despite the strong association with serum ALT activity and treatment regimen. This is not unexpected as we have already shown that serum MCP-1 concentration may be a reliable marker of inflammation in hepatic derangements in patients with chronic liver disease, a characteristic that is not shared by serum CRP concentration [28]. As previously described [29], HCV co-infection increased serum IL-8 levels with respect to healthy individuals. Surprisingly, our comparison did not reach statistically significance, suggesting that IL-8 liver expression could be partially affected by antiretroviral HIV therapy as shown in Figure 2D. Moreover, the correlation between serum insulin and serum FASN was maintained irrespective of HCV co-infection or altered ALT values in HIV-infected patients.

Abnormalities of glucose regulation, including impaired glucose tolerance and insulin resistance, are often developed among HIV-infected patients, and co-infection with HCV appears to exacerbate insulin resistance in these patients [30-33]. Insulin resistance in this population may result from antiviral medication, effects of HIV and/or HCV *per se*, or from other indirect effects, such as fat redistribution. Considering that increased MCP-1 forms also a vicious adipokine network causing insulin resistance and metabolic syndrome [34], both the chronic viral state itself and the host immune response can give rise to glucose and lipid metabolic disorders which, in turn, are risk factors for hepatic damage. Therefore, it is tempting to suggest that molecular determinants of HIV/HCV action on FASN release are closely linked to the overall metabolic regulation. In this scenario, increased concentrations of serum FASN might universally occur in metabolic disorders in which insulin resistance could be prominent. Supporting this notion, we and others have found that serum FASN concentration is increased in patients with non-alcoholic steatohepatitis or chronic liver impairment [35,36]. We have also found higher concentrations of circulating FASN in patients with type 2 diabetes [9]. Administration of insulin sensitizers significantly prevented FASN release from cultured human adipose tissue explants whereas treatment with inflammatory stimuli increased the amount of extracellular FASN [9]. We now add chronic infection with HIV/HCV as a novel pro-insulin resistance setting in which serum FASN concentration is significantly increased.

We have also provided experimental evidence to suggest that FASN release is an active and controlled process through the activation of AMP-activated protein kinase (AMPK) [15], a key enzyme that regulates the body energy balance [37-39]. We acknowledge that the ultimate mechanisms through which HIV/HCV infections influence the FASN release cannot by addressed by our study but HIV/HCV-induced changes on AMPK function might play a pivotal role. HIV and HCV are slow-growing viruses that should maintain beneficial host cell functions for an extended period as signs of disease do not usually show up until months or years after initial exposure. This requires a strict control of cellular AMPK because intense response and energy utilization could be deleterious to the viral infection [40]. We hypothesize that upon AMPK activation, the extracellular release of excess FASN may provide a rapid mechanism to prevent further energy consumption. Conversely, if we assume that FASN up-regulation is important in the pathogenesis of HIV and HCV infection, as it has been already demonstrated in infections by the hepatitis B virus and the coxsackievirus B3 [41], it is conceivable that FASN might be a potential therapeutic target for an antiviral therapy.

## Conclusions

Serum FASN concentration is significantly increased in HIV-infected patients. The release of the extracellular form of FASN into the circulation is further enhanced in patients who are co-infected with HCV or are not undergoing antiretroviral therapy. Subsequent studies should explore the usefulness of serum FASN concentration measurement to monitor the effects viral infections on disease progression and survival as well as the putative prognostic or diagnostic role for patients in whom repetitive viral infections and associated diseases are expected.

## Abbreviations

ALT: alanine aminotransferase; AMPK: AMP-activated protein kinase; BMI: body mass index; CRP: C-reactive protein; FASN: fatty acid synthase; HCV: hepatitis-C virus; IL-6: interleukin-6; IL-8: interleukin-8; MCP-1: monocyte chemoattractant protein-1; NEFAs: non-esterificated fatty acids; NNRTI: non-nucleoside reverse transcriptase inhibitors.

## Competing interests

The authors declare that they have no competing interests.

## Authors' contributions

GA, CAV, JAM, and JJ drafted the manuscript, participated in the design and coordination of the study. GA, COF, RBD, AR and FRS performed experimental procedures and collected data regarding HIV-infected patients. GA and CAV performed the statistical analysis. AVM and JC commented on the manuscript and participated in the design. All authors read and approved the final manuscript.

## Pre-publication history

The pre-publication history for this paper can be accessed here:

http://www.biomedcentral.com/1471-230X/10/92/prepub
